# A novel decarboxylating amidohydrolase involved in avoiding metabolic dead ends during cyanuric acid catabolism in *Pseudomonas* sp. strain ADP

**DOI:** 10.1371/journal.pone.0206949

**Published:** 2018-11-06

**Authors:** Lygie Esquirol, Thomas S. Peat, Matthew Wilding, Carol J. Hartley, Janet Newman, Colin Scott

**Affiliations:** 1 Biocatalysis and Synthetic Biology Team, CSIRO Land & Water, Canberra, ACT, Australia; 2 Research School of Chemistry, Australian National University, Canberra, ACT, Australia; 3 CSIRO Biomedical Manufacturing, Parkville, Melbourne, VIC, Australia; 4 Synthetic Biology Future Science Platform, CSIRO Land & Water, Canberra, ACT, Australia; Karl-Franzens-Universitat Graz, AUSTRIA

## Abstract

Cyanuric acid is a common environmental contaminant and a metabolic intermediate in the catabolism of *s-*triazine compounds, including atrazine and other herbicides. Cyanuric acid is catabolized *via* a number of bacterial pathways, including one first identified in *Pseudomonas* sp. strain ADP, which is encoded by a single, five-gene operon (*atzDGEHF*) found on a self-transmissible plasmid. The discovery of two of the five genes (*atzG* and *atzH*) was reported in 2018 and although the function of *atzG* was determined, the role of *atzH* was unclear. Here, we present the first *in vitro* reconstruction of the complete, five-protein cyanuric acid catabolism pathway, which indicates that AtzH may be an amidase responsible for converting 1,3-dicarboxyurea (the AtzE product) to allophanate (the AtzF substrate). We have solved the AtzH structure (a DUF3225 protein from the NTF2 superfamily) and used it to predict the substrate-binding pocket. Site-directed mutagenesis experiments suggest that two residues (Tyr22 and Arg46) are needed for catalysis. We also show that *atzH* homologs are commonly found in Proteobacteria associated with homologs of the *atzG* and *atzE* genes. The genetic context of these *atzG-atzE-atzH* clusters imply that they have a role in the catabolism of nitrogenous compounds. Moreover, their presence in many genomes in the absence of homologs of *atzD* and *atzF* suggests that the *atzG-atzE-atzH* cluster may pre-date the evolution of the cyanuric acid catabolism operon.

## Introduction

The symmetrical triazine cyanuric acid (1,3,5-triazine-2,4,6-triol) is a common anthropogenic compound that is used in the synthesis of a variety of disinfectants (e.g., trichlorocyanuric acid) and herbicides (e.g., atrazine). It is catabolized and used as a nitrogen source by a number of bacteria and can be mineralized to carbon dioxide and ammonia [1]. Cyanuric acid is also a common intermediate in the bacterial metabolism of other *s-*triazine compounds such as melamine, atrazine and simazine [1].

**Fig 1:**
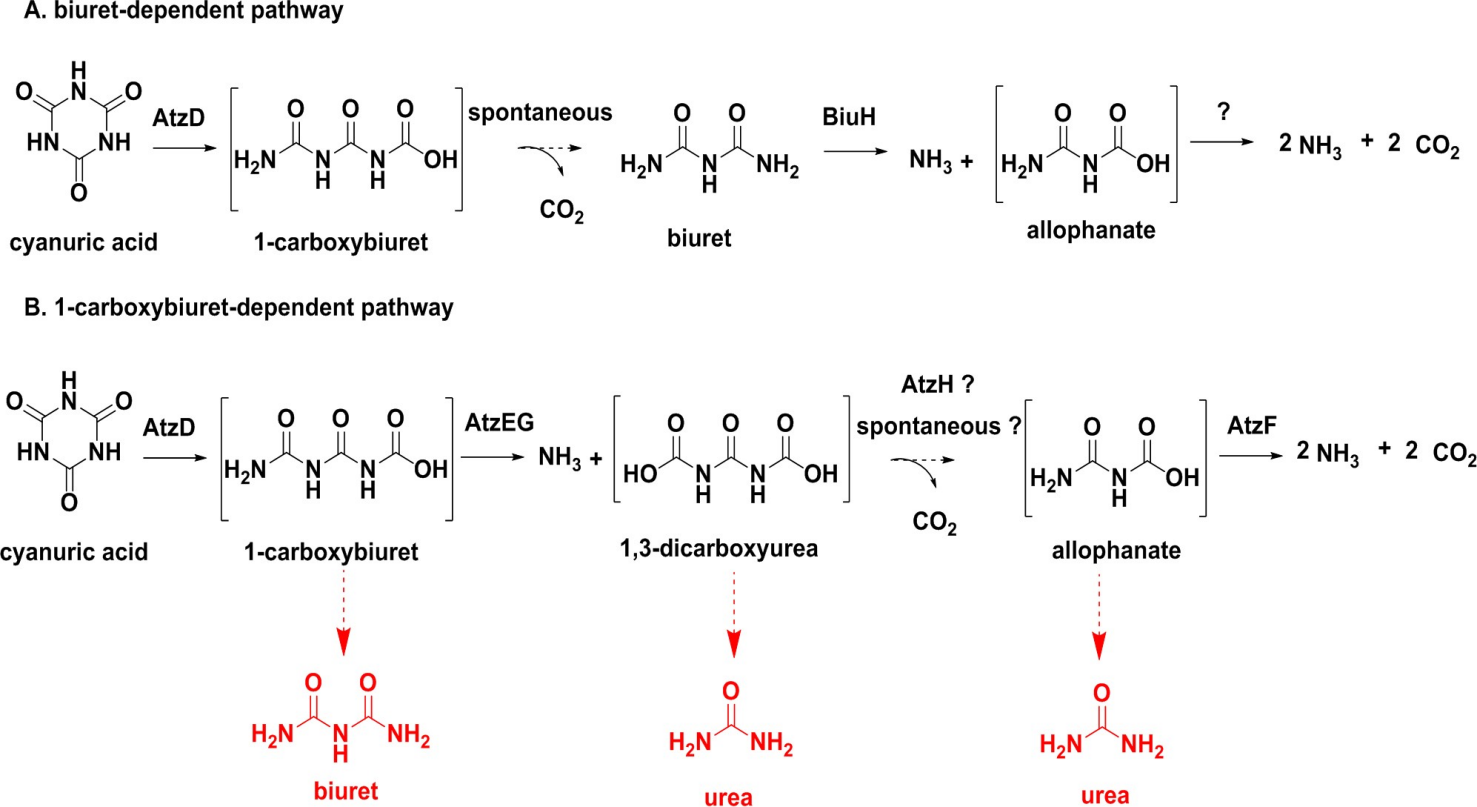
Bacterial cyanuric acid catabolism. A. The 1-carboxbiuret-dependent pathway first described in *Pseudomonas* sp. ADP; and, B. the biuret-dependent degradation pathway described in *Rhizobium leguminosarum* bv. *viciae* 3841. Compounds in square brackets are unstable under physiological conditions: the terminal amides hydrolyze to liberate CO_2_.

There are at least two pathways for the mineralization of cyanuric acid, a biuret-dependent pathway first described in *Rhizobium leguminosarum* bv. *viciae* 3841 (Fig 1A) [2,3], and a 1-carboxybiuret-dependent pathway observed in *Pseudomonas* sp. strain ADP (Fig 1B) [4,5]. The *Rhizobium leguminosarum* bv. *viciae* 3841 pathway employs cyanuric acid amidohydrolase (E.C. 3.5.2.15)[2,6,7] to ring-open cyanuric acid and the product (1-carboxybiuret) then undergoes solvent-mediated hydrolysis under physiological conditions to form biuret. Biuret is deaminated by biuret amidohydrolase (E.C. 3.5.1.84) to form allophanate and ammonia. The genes encoding the cyanuric acid hydrolase (E.C. 3.5.2.15) and biuret hydrolase (E.C. 3.5.1.84) are co-located on a large plasmid and may form an operon (although this has not been demonstrated). The fate of allophanate produced in this pathway remains unknown, although there are several genes in the *Rhizobium leguminosarum* bv. *viciae* 3841 genome that could degrade allophanate or its autohydrolysis product, urea [2,3].

The *Pseudomonas* sp. strain ADP cyanuric acid mineralization pathway is encoded by a single, five-gene operon: *atzDGEHF* [8]. The *atzG* and *atzH* genes were discovered recently and have been shown to encode proteins that are functionally expressed in *Pseudomonas* sp. strain ADP [8]. AtzG is non-catalytic and forms an obligate heterotetramer with the 1-carboxybiuret amidohydrolase, AtzE. In the absence of AtzG, AtzE is not soluble. The AtzEG complex is thought to have evolved from the GatCA component of the bacterial transamidosome (GatCAB) [8]. Like the *Rhizobium leguminosarum* bv. *viciae* 3841 pathway, *Pseudomonas* sp. strain ADP also ring opens cyanuric acid using cyanuric acid amidohydrolase (AtzD) [7,9]. In this pathway, it is 1-carboxybiuret that is deaminated by the next enzymatic step, which is catalyzed by 1-carboxybiuret amidohydrolase (AtzE) to form 1,3-dicarboxyurea [8]. 1,3-Dicarboxyurea must then undergo a single decarboxylation step to deliver allophanate to AtzF, which is again deaminated to ultimately produce ammonia and carbon dioxide [10–13].

Several of the metabolic intermediates in the 1-carboxybiuret-dependent pathway are unstable under physiological conditions: 1-carboxybiuret forms biuret and CO_2_, 1,3-dicarboxyurea and allophanate both form urea and CO_2_, and dicarboxyammonia (from allophanate deamination) forms ammonia and CO_2_. *Pseudomonas* sp. strain ADP is unable to degrade biuret or urea, and these solvent-mediated reactions effectively form metabolic ‘dead-ends’ [2,10]. Presumably, the enzyme-catalyzed reactions have rates that are sufficient to avoid this outcome, effectively using the enzyme-mediated rate enhancement to control which hydrolyses occur. In this context, it is perhaps counter-intuitive that the conversion of 1,3-dicarboxyurea to allophanate would be uncontrolled, which would potentially lead to urea accumulation *via* the solvent-mediated process; however, to date no enzyme has been identified that catalyzes this step [8].

Previously, proteomic analysis had demonstrated that AtzH is expressed from the cyanuric acid catabolism operon. However, its function had not been explored in previous studies [8]. Herein, we present the structure of AtzH and explore its potential function.

## Methods and materials

### Cloning and mutagenesis

The *AtzH* gene, coding for the protein WP_006390399.1 (KSW21442.1), was cloned directly from *Pseudomonas* sp. strain ADP genomic DNA with appropriate primers (5’ to 3’: CGACGACATATGCTCGAGATGCAAATTAATCTACC and TGCTGCGAGCTCCCTAGGTCAGGAAACGGGC), before being subcloned into the *Nde*I and *Bam*HI sites of the pETcc2 [9], which enabled production of AtzH with an N-terminal hexa-his-tag with thrombin cleavage site (S1 Fig). Cloning of the other genes used in this work has been described elsewhere [9,12,13].

The AtzF Ser189Ala variant and fourteen variants of AtzH (Tyr22Ala, Tyr22Phe, Arg46Ala, Arg46Lys, Arg63Ala, Arg63Lys, Arg66Ala, Arg66Lys, Arg73Ala, Arg73Lys, Arg96Ala, Arg96Lys, Gln106Ala, Gln108Ala) were produced using the QuikChange Lightning Multi Site-Directed mutagenesis kit from Agilent Technologies (Santa Clara, CA) with the mutagenic primers listed in S1 Table.

### Heterologous protein expression and purification

The expression vectors were used to transform *Escherichia coli* BL21 (λDE3) cells (Invitrogen). Bacteria were grown on Luria-Bertani (LB) medium containing ampicillin 100 μg/mL for the pETcc2 constructs or chloramphenicol 34 μg/mL for the pACYCDuet-1 construct (used to co-express AtzE and AtzG). Cells were grown with shaking at 200 rpm at 28°C. Protein expression was induced at an OD_600_ of 0.8 by addition of isopropyl-β–D-1-thiogalactopyranoside (IPTG; 1 mM final concentration).

Cells were harvested 24 hours after induction by centrifugation at 5000 x *g* for 15 minutes using an Aventi J-E centrifuge (Beckman Coulter, Indianapolis, USA), resuspended in lysis buffer (25 mM potassium phosphate, 5 mM imidazole, pH 7.5) and lysed by passage through a Microfluidics homogenizer M-110P (Massachusetts, USA) five times at 15000 PSI. The lysis was followed by centrifugation at 18000 x *g* for 45 minutes to pellet the cellular debris, and the soluble fraction was used for further purification.

The soluble fraction was syringe filtered through a 0.22 μm filter (Merck Millipore, USA). The His_6_-tagged proteins were isolated from the filtrate using a 5 mL Ni-NTA HisTrap HP (Healthcare Life Sciences) with an imidazole gradient from 5 mM to 500 mM over 10 column volumes (CV). SDS-PAGE gel analysis was performed to assess the purity of the fractions.

Fractions containing AtzH were pooled and concentrated to 8 mg/mL for crystallography using an Amicon Ultra-15 centrifugal filter unit. Finally, size exclusion chromatography was performed using a 130 mL column packed with Superdex 76 preparation grade resin (GE Healthcare Life Sciences), equilibrated with 25 mM HEPES and 200 mM NaCl, pH 7.5, over 1.5 CV.

All chromatography steps were performed using an ÄKTA purifier UPC 10 (GE Healthcare Life Sciences).

### Differential Scanning Fluorimetry (DSF)

Initially, buffer screening by DSF was performed on the wild-type protein–thirteen different combinations of salt/buffer at different pHs were tested in triplicate (Buffer screen 9 protocol)[14]. Protein was tested at 8 mg/mL in gel filtration buffer; 300 nL protein was diluted into 19.4 μL of each buffer, and 300 nL of a 1:20 dilution of sypro orange (Sigma S5692) was added. The temperature was increased from 20 to 100°C in increments of 0.5°/ 5 sec. This gave a *T*_m_ of 55.8 +/- 0.1°C in the gel filtration HEPES buffer. A slight improvement was seen when the protein was in 50 mM Tris pH 8, 200 mM NaCl (*T*_m_ of 57.1 +/- 0.1°C). The fourteen mutants were also tested for stability using DSF. Each sample was diluted to 1 mg/mL in 50 mM HEPES pH 7.5, 200 mM NaCl, and 1 μL of protein was added to 19 μL of mix of 4 mL 50 mM HEPES pH 7.5, 200 mM NaCl, 2.5 μL Sypro Dye. Each variant was run in 6-fold replicates, and compared to the wild-type protein which was included as a control (under these conditions the wild type protein had a *T*_m_ of 55.0 +/- 0.1°C). A complete table of the results is shown in S2 Table.

### Crystallography

Purified AtzH at 8 mg/mL protein was used to set up a PCT test (Pre-Crystallization Test [15]), and immediately showed microcrystal formation in the PEG based condition of the PCT. The protein was diluted to 4 mg/mL and then set up in 3 initial PEG-rich screens (shotgun, PACT and C3_3; conditions available from c6.csiro.au [16]), in SD2 sitting drop plates (Molecular Dimensions, UK) with an equal volume of crystallant (150 nL protein plus 150 nL reservoir) at 20°C. Crystals appeared overnight in many conditions. Further PEG based screens (C3_1->C3_4, Morpheus_C3) were also set up, with the protein at 4 mg/mL with and without added thrombin. Crystals continued to appear in many PEG-based conditions for up to 3 weeks after setting up the plates. The AtzH wild-type protein and the variants were also set up in co-crystallization trials with the PPDi inhibitor. 1 μL of a 40 mM solution of the inhibitor in protein buffer (50 mM HEPES, 200 mM NaCl pH 7.5) was added to 40 μL of the 4 mg/mL protein. The variants were each set up in two screens (Shotgun, C3_3) at 4 mg/mL (if accessible) with and without added inhibitor and incubated at 20°C. The mutants in general crystallized poorly, and only a few variants gave crystals suitable for harvesting. Crystals were cryoprotected with addition of glycerol to a final concentration of 20% and cryo-cooled in liquid nitrogen prior to data collection. Crystals used in data collection were harvested directly from the screening plates.

The native AtzH P1 crystal form crystal was harvested from a 300 nL drop consisting of a 1:1 ratio of protein and reservoir, where the reservoir consisted of 200 mM ammonium acetate, 30% PEG 4000 and 100 mM sodium acetate buffer pH 5.0. The native C222_1_ crystal form was harvested from a 300 nL drop consisting of a 1:1 ratio of protein and reservoir, where the reservoir contained 20% PEG 6000, 2.5% v/v tert-butanol with 100 mM sodium citrate buffer at pH 5.5.

The R73K mutant (which crystallized in space group P2_1_) was harvested from a 300 nL drop consisting of a 1:1 ratio of protein/inhibitor and reservoir, where the reservoir contained 0.2 M diammonium tartrate, 20% w/v PEG 3350. Data sets were obtained at the Australian Synchrotron beamline MX2 using a Dectris Eiger 16M detector and a full 360 degrees of data were taken. The data were indexed using DIALS [17–21], scaled using Aimless [22] and the original structure solution was determined by molecular replacement using Phaser [20,21] with PDB code 2RCD as the model, after sequence alignment with Clustal-Omega and pruning using Chainsaw [23,24] to give an appropriate starting model. The model was rebuilt manually using Coot [25] and refined using Refmac [24,26].

*In silico* substrate docking was done using Accelrys Discovery Studio (v4.1). The Define and Edit Binding Site program was used to identify cavities in AtzH. Models of 1,3-dicarboxyurea were prepared using the same client in doubly protonated, deprotonated and singly protonated states and docked into the cavity identified previously using the CDOCKER program with default parameters.

### Enzyme assays

Reaction rates were monitored by detecting the production of ammonium in μmoles per second using the glutamate dehydrogenase GDH-coupled assay, described previously [3]. GDH catalyzes the NADH-dependent amination of alpha-ketoglutarate. Ammonia production was followed using the decrease of absorbance by UV spectrophotometry at 340 nm, due to the oxidation of NADH by GDH. 1.25 U of GDH was used in a 250 μL reaction volume, the final concentrations of alpha-ketoglutarate and NADH were 3.5 mM and 0.2 mM, respectively. The buffer was 25 mM potassium phosphate, pH 8.5. Cyanuric acid hydrolysis by AtzD was followed by UV spectrophotometry at 214 nm [13]. In all the reaction mixes, 12 μM of AtzD, 0.17 μM of AtzE, 0.17 μM AtzF and 3.3 μM of AtzH or its variants were used in presence of 1 mM of cyanuric acid.

## Results and discussion

### Structural characterization of the *Pseudomonas* sp. strain ADP AtzH

We obtained the X-ray crystal structure of the AtzH. His_6_-tagged AtzH was produced in *E*. *coli* and purified, yielding 5 mg of AtzH per 1 L of culture. Size exclusion chromatography performed on AtzH after removal of the his-tag, suggested that the native protein had a molecular mass of ~ 29 kDa, consistent with a dimer (2 x 14,655 kDa) (S2 Fig). Differential Scanning Fluorimetry (DSF) was used to estimate the stability of the protein and showed that the native protein had a *T*_m_ of 57.2°C. Well diffracting crystals of AtzH grew readily, and two different space groups were observed: P1 and C222_1_. X-ray diffraction data were collected for both.

**Fig 2:**
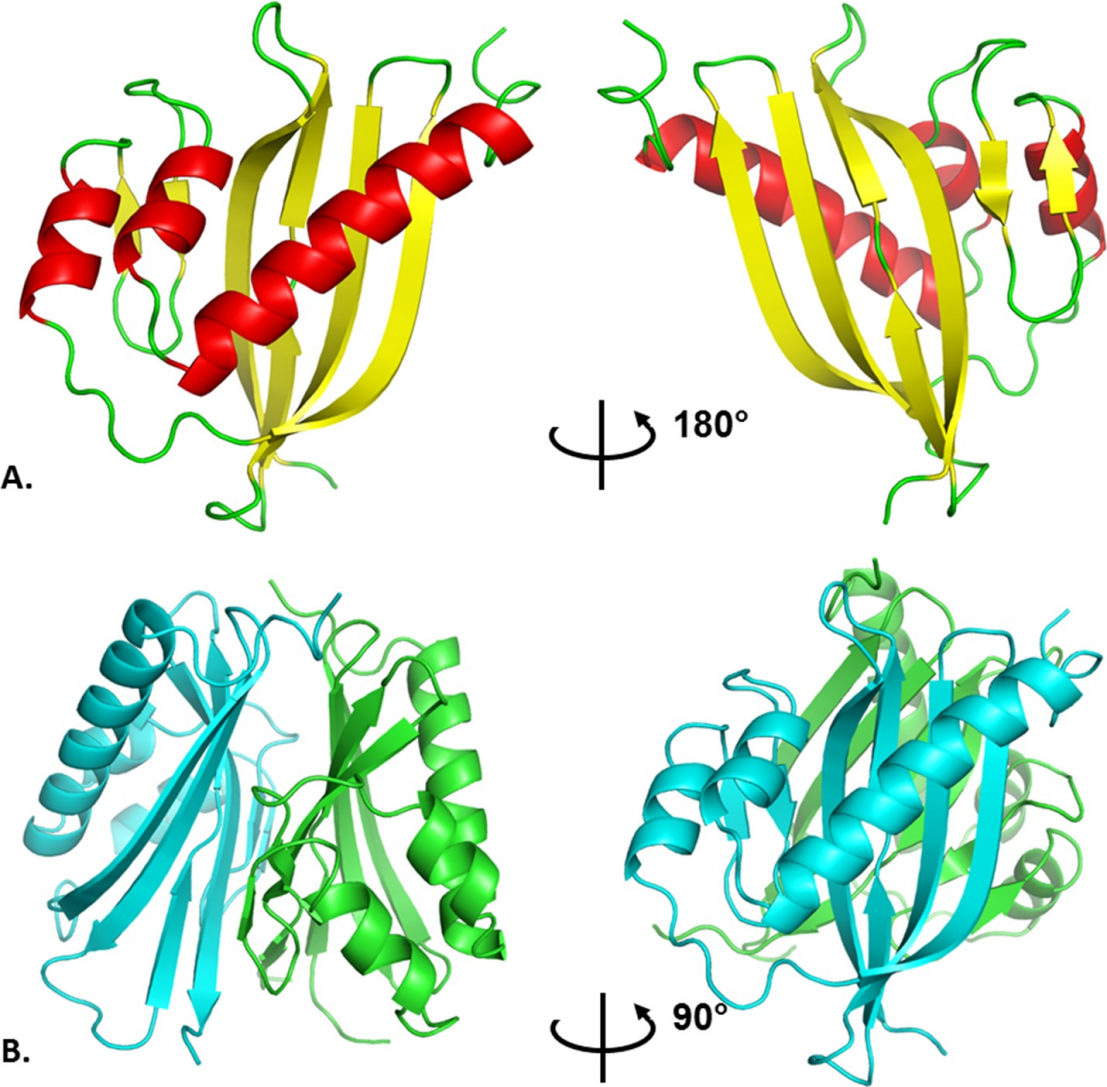
The AtzH structure. A) The AtzH monomer with the seven-stranded anti-parallel β-sheet highlighted in yellow and the three helices are shown in red; B) The AtzH dimer with the two protomers interacting through the β-sheet secondary structure with the helices sitting on the outside. The two anti-parallel β-sheets sit at an approximate 30 degree angle to each other.

The structure of AtzH was solved by molecular replacement, using the structure of a functionally uncharacterized protein from *Pectobacterium atrosepticum* strain SCRI 1043 (PDB accession number: 2RCD) [27] as the model. 2RCD is 56% identical to AtzH and there is a 1.0 Å rmsd between Cα atoms when superposing the structures (x-ray data are in Table 1). The structures exhibit minor differences at the N- and C-termini and in some loop regions (including the loop with weak density in AtzH, residues 66 to 73). Additionally, 2RCD has a dimer in the asymmetric unit, which mirrors the dimer seen in AtzH where two, six-stranded β-sheets sit face-on at an angle to form the interface. One long helix and two short helices sit on the outside of this interface and the overall dimer structure is very compact (Fig 2). Analysis with PISA [28] gives an interfacial surface area of almost 3,200 Å^2^ (or about 29% of the total surface area of the protein).

**Table 1:** Crystal structure parameters.

	6BJU Wild-type	6C6G Wild-type	6D63 Arg73Lys
**Data collection**			
Space group	P1	C2221	P21
Cell dimensions			
*a*, *b*, *c* (Å)	51.8, 51.8, 59.7	71.5, 75.0, 96.2	99.7, 74.7, 103.6
α, β, γ (°)	99.8, 102.2, 92.5	90, 90, 90	90, 96.7, 90
Resolution (Å)	1.64 (1.67–1.64)	1.80 (1.84–1.80)	2.30 (2.35–2.30)
*R*_merge_	0.087 (0.674)	0.159 (1.581)	0.267 (1.402)
*R*_*pim*_	0.054 (0.412)	0.045 (0.438)	0.109 (0.561)
*I* / σ*I*	7.8 (1.9)	11.2 (2.2)	6.6 (1.6)
*CC1/2*	0.995 (0.733)	0.998 (0.837)	0.986 (0.761)
Completeness (%)	97.2 (95.3)	100 (100)	100 (100)
Redundancy	3.6 (3.7)	13.3 (13.7)	6.9 (7.1)
**Refinement**	2 dimers	1 dimer	6 dimers
Resolution (Å)	50.5–1.64	37.5–1.80	50.1–2.30
Unique reflections	67,428	23,104	64,253
Rwork / Rfree	19.9 / 22.8	16.2 / 20.6	24.9 / 28.7
No. atoms	4538	2307	12,009
Protein	4029	2110	11,791
Inhibitor	n/a	n/a	30
Water	509	178	288
B-factors (Å2)	21.6	26.2	25.3
Protein	21.7	26.6	26.4
Inhibitor	n/a	n/a	40.9
Water	31.4	31.1	17.2
R.m.s deviations			
Bond lengths (Å)	0.020	0.017	0.011
Bond angles (°)	1.856	1.677	1.363

Note: Values in parenthesis are for the highest resolution shell.

AtzH belongs to NTF2-fold superfamily, which contains 38 protein families [29], including the AtzH family: DUF3225 (DUF: domain of unknown function). Proteins of the NTF2 superfamily fulfil a number of functions, including enzymatic functions such as limonene-1,2-epoxide hydrolase, scytalone dehydratase, δ5-3-ketosteroid isomerase and polyketide cyclase [30–33] and non-enzymatic roles including nuclear transport and bacterial secretion [34–39]. Among the 170 protein structures belonging to NTF2-fold superfamily available on the PBD database, 24 are assigned as DUF3225 [40]; no DUF3225 protein had been functionally characterized prior to this study.

### A physiological role for AtzH

**Fig 3:**
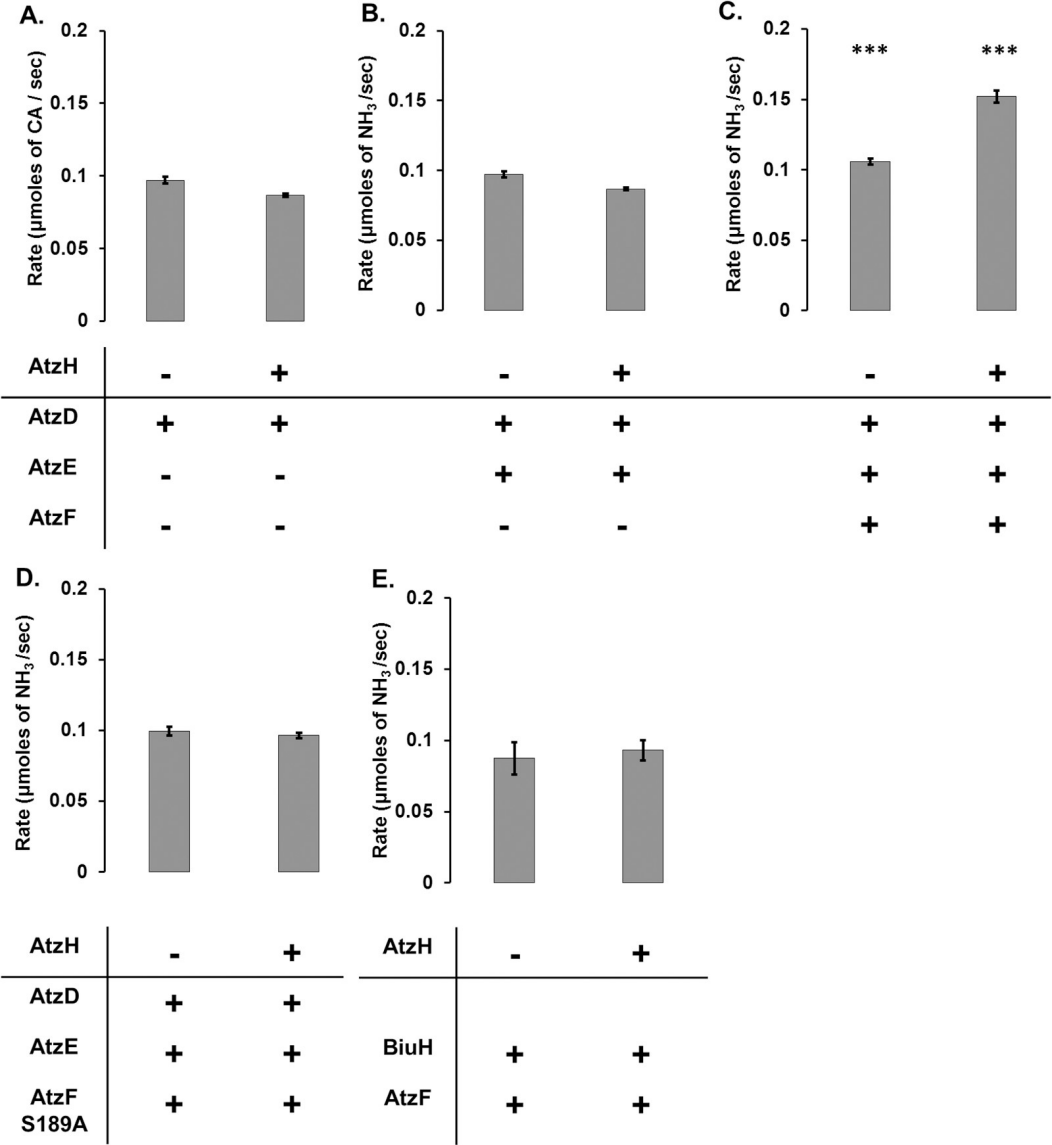
Impact of AtzH on the rate of the cyanuric acid pathway *in vitro*. A) Rate of cyanuric acid (CA) degradation in presence of AtzD with/without AtzH; B) Rate of ammonia production when CA is put in presence of AtzD and AtzEG with/without AtzH; C) Rate of ammonia production when CA is put in presence of AtzD, AtzEG and AtzF with/without AtzH; D) Rate of ammonia production when CA is put in presence of AtzD, AtzEG and an inactive variant of AtzF Ser189Ala with/without AtzH; E) Rate of ammonia production when biuret is put in presence of BiuH and AtzF with/without AtzH. *** indicates a significant difference between the rate measured in the presence of AtzH and the rate measured without the AtzH (pval < 0.005, measured by Student’s t-test; n = 3).

We investigated the effect of AtzH on the cyanuric acid catabolic pathway by reconstituting the complete cyanuric acid mineralization pathway *in vitro* using purified enzymes; i.e., AtzD, AtzEG and AtzF with and without AtzH. Inclusion of AtzH led to an increased rate of ammonia production from cyanuric acid by the pathway (Fig 3C). This is consistent with an enzymatic role for AtzH, potentially fulfilling the role of a decarboxylating 1,3-dicarboxyurea amidohydrolase. Unfortunately, both the substrate and product for the putative AtzH-mediated reaction are unstable under the conditions required for enzyme function (i.e., aqueous buffer at physiological pH) and so the reaction could not be followed directly. However, it was possible to probe the role of AtzH by varying the composition of the *in vitro* pathway.

The AtzD substrate (cyanuric acid) is stable and its hydrolysis easily followed by measuring the reduction in absorbance at 214 nm caused by AtzD-mediated ring-opening [9]. It was therefore possible to directly test the influence of AtzH on this step of the pathway by incubating AtzD and its substrate in the presence or absence of AtzH, and AtzH did not influence the rate of this step (Fig 3A). The influence of AtzH on the AtzEG-catalyzed reaction was also investigated (Fig 3B). The substrate for AtzEG (1-carboxybiuret) is unstable and was therefore produced *in situ via* AtzD-mediated ring opening of cyanuric acid. One product of the AtzEG reaction is ammonia, which was used to determine the rate of the reaction through a GDH-coupled assay. *In vitro* cascades containing AtzD and AtzEG evolved ammonia from cyanuric acid at the same rate whether or not AtzH was included in the incubation (Fig 3B).

These observations suggest that the AtzH-dependent increase in the rate of ammonia production from the pathway was also AtzF-dependent; i.e., AtzH likely increases the rate at which AtzF produces ammonia, possibly by increasing the rate at which allophanate is fed to AtzF. To further investigate this, an *in vitro* pathway comprised of AtzD, AtzEG and a variant of AtzF that lacked the active-site nucleophile (AtzF Ser189Ala) was also tested. In this reaction, the presence of AtzH also failed to improve the rate of ammonia production (Fig 3D), confirming that the AtzH-mediated effect observed in the fully reconstituted pathway is dependent on the presence of active AtzF. These observations imply that AtzH is either increasing the rate of allophanate production from 1,3-dicarboxyurea, or enhancing the activity of AtzF itself.

To test whether AtzH increases the catalytic activity of AtzF, an alternative *in vitro* pathway was tested in which biuret was incubated with the biuret amidohydrolase BiuH [2] and AtzF. BiuH produces allophanate directly, and so an increase in the rate of ammonia production in this pathway would indicate that AtzH is an allosteric activator of AtzF. However, the presence of AtzH made no measurable difference to the rate of ammonia production (Fig 3E), suggesting that AtzH does not ‘activate’ AtzF. The simplest explanation for these observations is that AtzH mediates the decarboxylation of 1,3-dicarboxyurea, the product from AtzEG, to form allophanate, the substrate for AtzF.

### Defining the active site of AtzH

**Fig 4:**
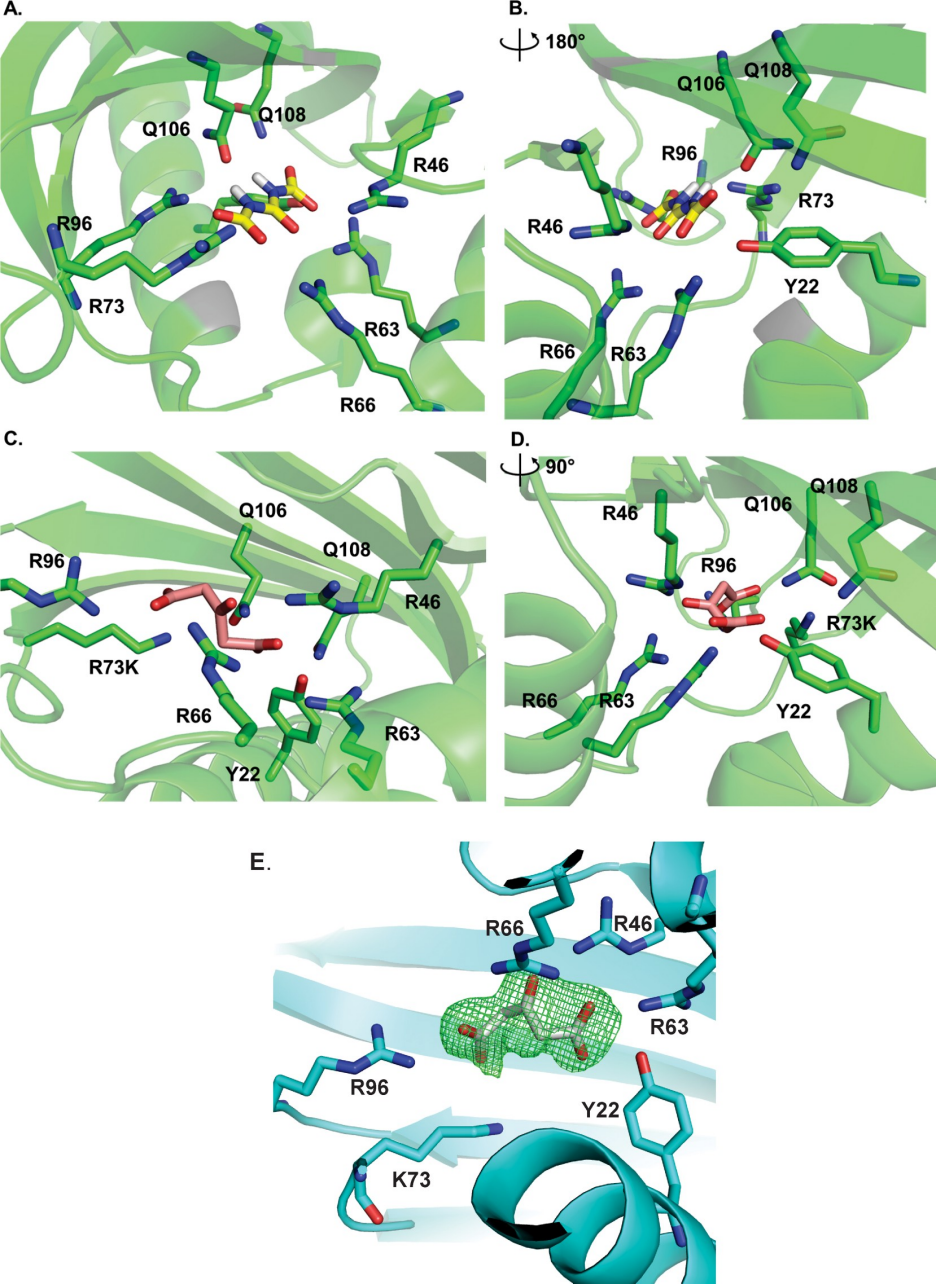
Substrate and substrate analog binding by AtzH. A-B) Simulated binding of 1,3-dicarboxyurea (yellow) by AtzH. Amino acids residues involved in binding 1,3-dicarboxyurea are shown as green sticks; C-D) X-ray structure of AtzH Arg73Lys co-crystalized with the substrate analog 1,3 dicarboxyacetone (pink). The distance between Tyr22 and the carbon of the carboxyl group is <3 Å; E) Polder map (3 sigma density contour) of 1,3 dicarboxyacetone bound to the AtzH Arg73Lys variant.

The AtzH active site was sought by examining the X-ray structure. As 1,3-dicarboxyurea is unstable, it was not possible to produce crystals of AtzH in its presence. However, an *in silico* docking simulation between the X-ray crystal structure of the AtzH dimer and 1,3-dicarboxyurea elucidated a potential active site (Fig 4A and 4B). The putative substrate bound with good complementarity to an arginine-rich pocket comprised of Tyr22, Arg46, Arg63, Arg66, Arg73, Phe94, Arg96, Gln106 and Gln108 (Fig 4A and 4B). The arginine residues form an extensive hydrogen bonding network with the carbonyl oxygens of 1,3-dicarboxyurea. Tyr22 is positioned within 3 Å of the carbonyl carbon of one of the two terminal carboxylic acids, and Arg46 is within hydrogen-bonding distance of the hydroxyl group of Tyr22. Tyr22 and Arg46 are therefore well positioned to form a catalytic dyad, albeit one of atypical composition.

**Fig 5:**
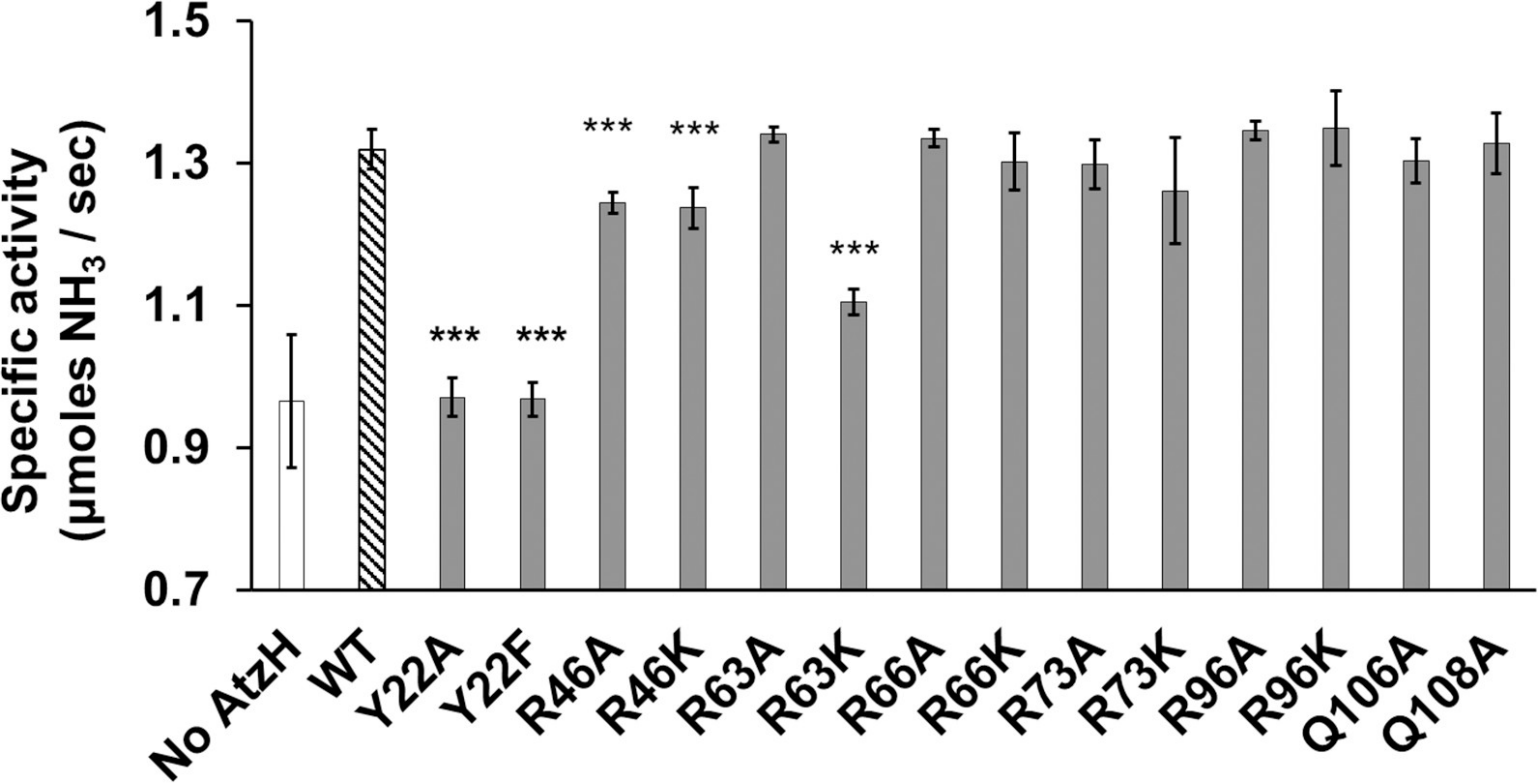
Impact of the presence of AtzH variants on the ammonium production rate of the *in vitro* cyanuric acid catabolism pathway. The rates of ammonia production by the cyanuric acid catabolism pathway (AtzD, AtzEG and AtzF) reconstituted *in vitro* with no AtzH (white), wild-type AtzH (striped) or an AtzH variant (gray) are shown. *** indicates an ammonia production rate that is significantly different from a reaction in the presence of wild-type AtzH (pval < 0.005, measured by Student’s t-test; n = 3).

Guided by the results of the docking experiment, fourteen variants of AtzH were produced: Tyr22Ala, Tyr22Phe, Arg46Ala, Arg46Lys, Arg63Ala, Arg63Lys, Arg66Ala, Arg66Lys, Arg73Ala, Arg73Lys, Arg96Ala, Arg96Lys, Gln106Ala, Gln108Ala (S1 Table). All of the variants were correctly folded, as assessed by DSF (S2 Table). Furthermore, X-ray structures of the R73K variant (PDB: 6D63) was also obtained and closely resembled the wild-type enzyme (PDB: 6BJT, 6BJU) except for the intended amino acid substitution. The AtzH variants were added to *in vitro* pathways comprised of AtzD, AtzEG and AtzF and incubated with cyanuric acid. Five of the variants (Tyr22Ala, Tyr22Phe, Arg46A, Arg46Lys and Arg63Lys) were found to enhance the rate of ammonia production, albeit to a significantly lower extent than the wild-type AtzH (Fig 5). The loss of function of Arg63Lys, but not of Arg63Ala, may suggest that Arg63 does not have a catalytic role. However, AtzH Tyr22Ala/Phe was indistinguishable from the AtzH-free cascade (Fig 5). The Arg46Ala and Arg46Lys variants both increased the rate of ammonia production by the cascade, but to a lesser degree than that of the wild-type AtzH (Fig 5). This may suggest that Tyr22 is the catalytic amino acid residue in AtzH, possibly acting as a proton donor (consistent with the catalytic role of tyrosine in other enzymes) [41], with Arg46 fulfilling a non-critical function As the substrate analog binds between Arg46 and Tyr22, it seems unlikely that Arg46 has a role in activating Tyr22 (noting that the substrate analog may not adopt exactly the same conformation as the bound substrate). However, this positioning of the active site residues relative to the bound analog is similar to that of the catalytic residues in inverting β-glycosidases that use an acid-base catalytic mechanism [42], and it is plausible that that a similar mechanism may be used here. Further work is needed to test this hypothesis.

The wild-type and variants were co-crystallized with 1,3-dicarboxyacetone, a stable structural analogue of 1,3-dicarboxyurea. In the AtzH Arg73Lys structure (PDB 6D63) 1,3-dicarboxyacetone was found to occupy the predicted active site of AtzH in a subset of the protomers (25%), indicating that this pocket is accessible by 1,3-dicarboxyurea-like small molecules (Fig 4C and 4D). The hydrogen bonding network is largely as predicted for 1,3-dicarboxyurea, and Tyr22 is well positioned as the catalytic residue.

### AtzH homologs in other bacterial genomes

AtzH is the first DUF3225 family protein that has been shown to possess a catabolic function, likely decarboxylating the product of AtzEG-mediated deamination of 1-carboxybiuret as part of the cyanuric acid mineralization pathway. Homologs of the other members of the pathway are found in different metabolic contexts: the AtzD homolog barbiturase participates in pyrimidine catabolism [43], the AtzEG homologs GatCA are involved with acyl-tRNA metabolism [44] and the AtzF homolog allophanate hydrolase is used in urea catabolism [45]. We were therefore interested to investigate whether AtzH homologs may also participate in alternative metabolic contexts.

**Fig 6:**
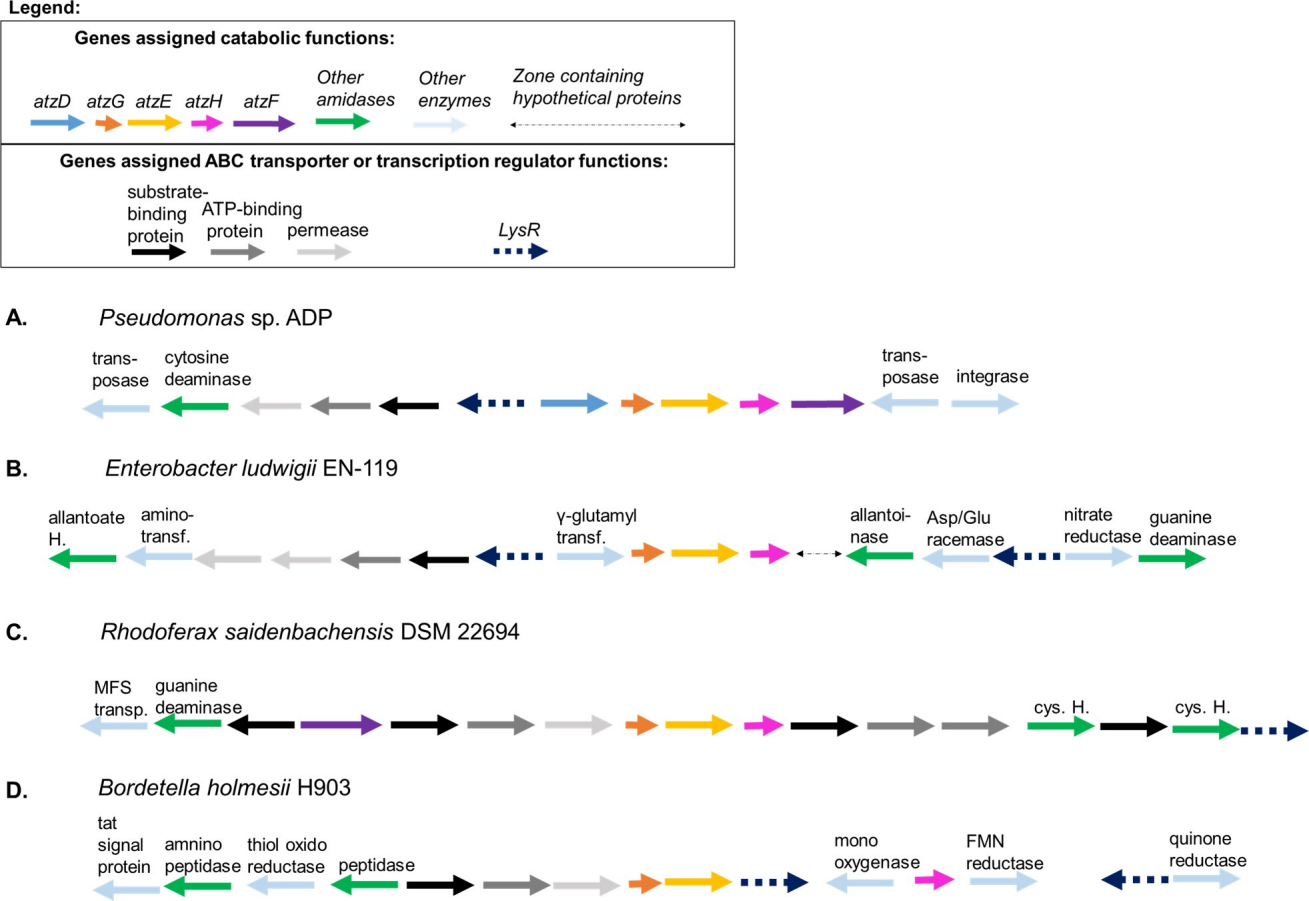
Representative genomic organization of *atzG-atzE-atzE* clusters in Proteobacteria. The organization of gene regions that contain the *atzG-atzE-atzH* clusters from A) *Pseudomonas* sp. strain ADP and, B-D) three bacteria that illustrate the most common genomic organizations associated with the cluster are shown. A comprehensive version is available (S3 Fig).

A thorough search of microbial genomes in the GenBank database revealed more than thirty examples of genomes containing homologs of *atzG*, *atzE*, and *atzH* organized as a single gene cluster with the same organization as that of the *Pseudomonas* sp. strain ADP cyanuric acid catabolism operon (Fig 6). Perhaps surprisingly, the *atzG*-*atzE*-*atzH* clusters were rarely associated with *atzD* or *atzF* homologs.

The most common arrangement, found in species of Enterobacteriaceae (*Pectobacterium*, *Serratia*, *Enterobacter*, *Klebsiella*, *Kosakonia*, *Pantoea*, *Yersinia* and *Erwinia*), has the *atzG*-*atzE*-*atzH* cluster immediately downstream of a gene predicted to encode γ-glutamyl transferase (*ggt*, E.C. 2.3.2.2). In these genomes, the *ggt-atzG*-*atzE*-*atzH* cluster is found downstream and on the opposite strand from a gene cluster encoding a LysR-family regulator, an ABC transporter, a transaminase and an allantoate amidohydrolase. The overall genetic organization of these clusters is similar to that of the cyanuric acid catabolism operon of *Pseudomonas* sp. strain ADP, albeit lacking *atzF* and with *atzD* replaced by *ggt*. (Fig 6B, S3 Fig)

**Fig 7:**
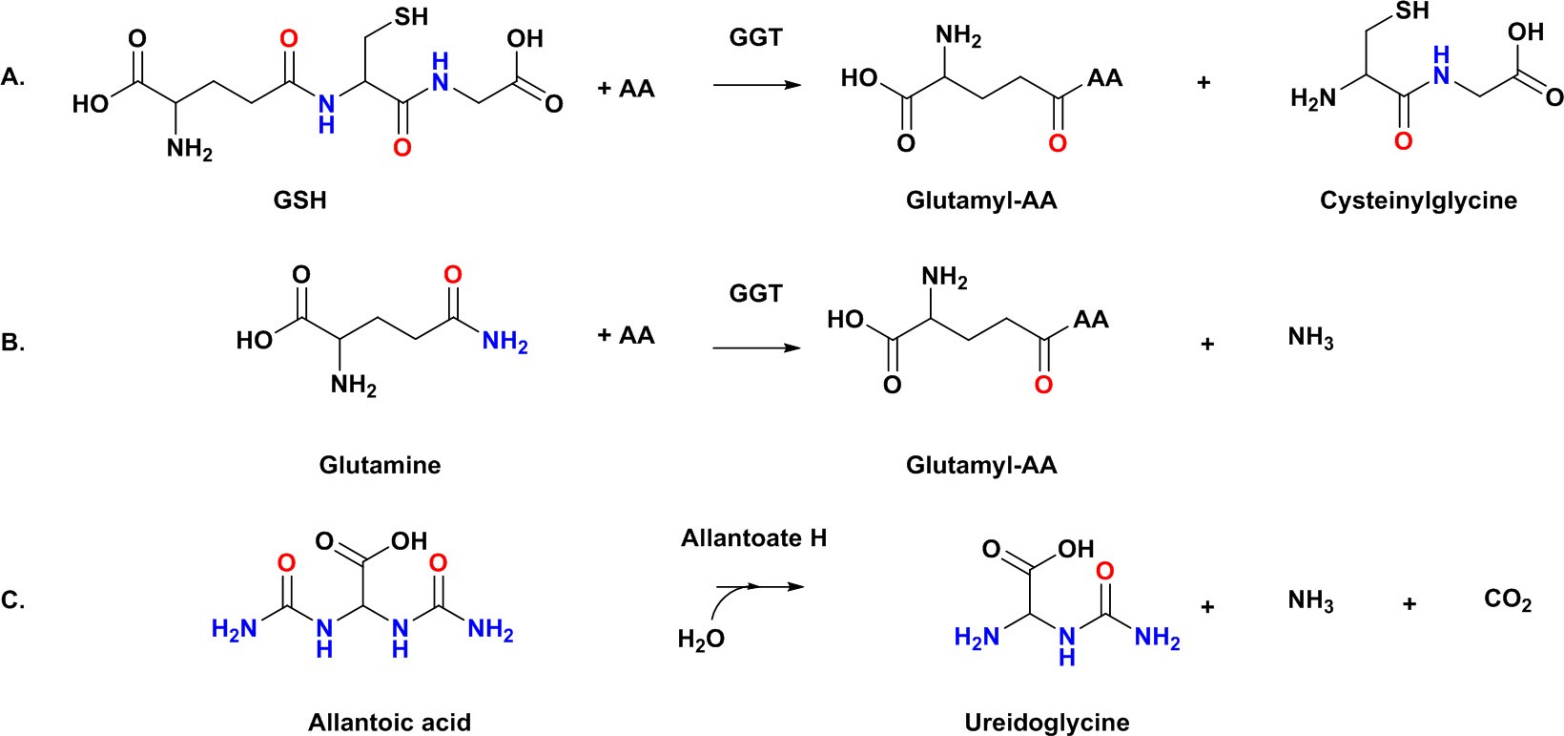
Hydrolyses catalyzed by enzymes encoded by genes associated with *atzG-atzE-atzH* clusters. A-B) Reactions performed by a gamma-glutamyltransferase (GGT) and C) an allantoate hydrolase. Ureido and amide groups are shown in colors.

γ-Glutamyl transferase, which also acts as glutathione hydrolase (E.C. 3.4.19.13), is responsible for the amide hydrolysis of glutathione to produce L-glutamate and L-cysteinylglycine (Fig 7). Further, the substrate for allantoate hydrolase, an enzyme found encoded in close proximity to the Enterobacteriaceae *atzG*-*atzE-atzH* clusters described here, contains two ureido groups (Fig 7) [46]. It is possible that *atzG*-*atzE-atzH* clusters form components of catabolic pathways in which the substrates possess amide groups.

There are also gene clusters found in β-proteobacter from the orders Comamonadacaea (*Rhodoferax* and *Variovorax*) and Burkholderiales (*Rhizobacter*, *Leptothrix*, *Methylibium* and *Rhizobiales*) that contain an allophanate hydrolase encoding gene. In all cases, the allophanate hydrolase gene is upstream of the *atzG-atzE-atzH* cluster and encoded on the same strand; in about half of the genomes there are genes encoding an ABC transporter separating the allophanate gene from the *atzG-atzE-atzH* cluster. Downstream, and encoded on the same strand as these *atzG-atzE-atzH* clusters, are genes encoding ABC transporters and amidase enzymes. This may indicate that these clusters are for the uptake and catabolism of nitrogenous compounds. (Fig 6C, S3 Fig)

Other Burkholderiales (*Bordetella* and *Achromobacter*) possess *atzG-atzE* and *atzH* clusters, but where the cluster also includes genes of unknown function that are annotated by homology as a transcriptional regulator, a monooxygenase and an FMN-reductase. In these clusters *atzH* is located between the genes encoding the monooxygenase and reductase, rather than immediately downstream of *atzG* and *atzE*. Finally, a number of diverse α-, β-, and γ-proteobacteria (*Pseudorhodoplanes*, *Methylobacterium*, *Thiomonas*, *Hydrogenophaga*, *Comamomonas*, *Pseudomonas*, and *Halothiobacillus*) have *atzG-atzE-atzH* clusters in unique genetic contexts, including the cyanuric acid catabolic operon of *Pseudomonas* sp. strain ADP. (Fig 6D, S3 Fig).

The occurrence of the *atzG-atzE-atzH* cluster appears to be far more wide-spread than the cyanuric acid catabolic pathway, and it is frequently associated with genes that may be involved in the catabolism of diverse nitrogen-containing compounds. However, the genetic contexts in which *atzH*-like genes are found provides little further evidence to suggest the specific function of the AtzH protein.

## Conclusion

The data presented here imply that AtzH is a decarboxylating, 1,3-dicarboxyurea amidohydrolase. As yet its catalytic mechanism is not known, but our results suggest it involves the action of Tyr22 and Arg46, and that these amino acid residues are positioned relative to the substrate in a manner consistent with an acid-base mechanism. AtzH also facilitates the only previously uncontrolled step in the catabolic pathway, and as such, we believe that we have now identified the last remaining enzyme in the *Pseudomonas* sp. strain ADP cyanuric acid catabolism pathway (Fig 1B). The pathway appears to be dependent on enzymatic steps that outcompete solvent mediated hydrolysis that would otherwise lead to the accumulation of dead-end metabolites (biuret and urea). With no endogenous biuret or urea hydrolases, formation of these metabolites effectively prevent access to two of the three nitrogen atoms of the triazine ring.

Unexpectedly, *atzG-atzE-atzH* -like clusters appear to be relatively common within the Proteobacteria. Given the amidase function of AtzE and proposed amidase activity of AtzH, it is perhaps unsurprising that the *atzG-atzE-atzH*-like clusters tend to be associated with genes predicted to be involved in nitrogen compound metabolism. Previously, we had demonstrated that *atzG* and *atzE* were likely to have co-evolved from components of the bacterial GatCAB (transamidosome) complex. Here, we report the discovery of *atzG-atzE-atzH*-like clusters associated with diverse genetic contexts and metabolic functions, implying that the formation of the *atzG-atzE-atzH* cluster may predate the evolution of the cyanuric acid catabolism operon.

## Supporting information

S1 Fig AtzH protein sequence. The AtzH sequence is shown in black and the supplementary hexa-his-tag with thrombin cleavage site in red.
(DOCX)

S2 Fig Purification of heterologous AtzH from *E*. *coli*. A. SDS-PAGE analysis of the fractions 2–11 of the Ni-NTA purification of His_6_-AtzH (left), UV absorbance trace of the Ni-NTA purification (right), fractions 7 to 11 were concentrated and further purified; B. SDS-PAGE analysis of the size exclusion, showing the content of fractions 7–11 (left); UV absorbance trace of the size exclusion (right). On the traces, the green line in A. represents the % buffer B, the blue line shows the UV absorbance at 280 nm in arbitrary units; M: molecular marker, W: whole cell, S: soluble.
(DOCX)

S3 Fig Genetic organization found flanking *atzE* homologues in other types of bacteria. A. Bacteria containing an allantoate hydrolase and glutamyl-transferase enzymes upstream of the *atzG*-*atzE*-*atzH* segment; B. Bacteria found to be containing an allophanate hydrolase homologue of AtzF found upstream of the *atzG*-*atzE*-*atzH* segment; C. Bacteria containing a non-adjacent AtzH homologue, located between a monooxygenase and reductase enzymes, downstream of the *atzG*-*atzE* segment; D. Bacteria presenting the *atzG*-*atzE*-*atzH* cluster within unique gene organizations. A legend is shown at the top of the figure. Green arrows represents amidase type enzymes, black and grey tones represent ABC transporters, discontinued dark blue arrows represent the presence of a *LysR* type regulator, genes encoding for AtzG, AtzE and AtzH homologues are shown by orange, yellow and pink arrows and light blue arrows represent other types of enzymes, respectively. This figure is not to scale.
(DOCX)

S1 Table Mutagenic primers used for introducing point mutation in AtzH’s sequence. (DOCX)

S2 Table DSF of the AtzH variants. (DOCX)
