# Supplementary material for: A novel decarboxylating amidohydrolase involved in avoiding metabolic dead ends during cyanuric acid catabolism in Pseudomonas sp. strain ADP
Source: PLoS One. 2018 Nov 6;13(11):e0206949. doi: 10.1371/journal.pone.0206949 (PMC6219798; doi:10.1371/journal.pone.0206949)
Supplement: S1 Fig — The AtzH sequence is shown in black and the supplementary hexa-his-tag with thrombin cleavage site in red. (DOCX) [file pone.0206949.s001.docx]

MGSSHHHHHHSSGLVPRGSHMQINLPEVHAEVTAQFVRYEKALTSNDTAVLNELFWNSPQTLRYGATENLYGYEAIAGFRATRSPNNLEREIVRTVITTYGHDFATANIEFRRLSHSQLTGRQSQTWMRTSQGWRVVAAHVSLIALPVS

**S1 Fig: AtzH protein sequence.** The AtzH sequence is shown in black and the supplementary hexa-his-tag with thrombin cleavage site in red.
