# Supplementary material for: A novel decarboxylating amidohydrolase involved in avoiding metabolic dead ends during cyanuric acid catabolism in Pseudomonas sp. strain ADP
Source: PLoS One. 2018 Nov 6;13(11):e0206949. doi: 10.1371/journal.pone.0206949 (PMC6219798; doi:10.1371/journal.pone.0206949)
Supplement: S2 Fig — A. SDS-PAGE analysis of the fractions 2–11 of the Ni-NTA purification of His6-AtzH (left), UV absorbance trace of the Ni-NTA purification (right), fractions 7 to 11 were concentrated and further purified; B. SDS-PAGE analysis of the size exclusion, showing the content of fractions 7–11 (left); UV absorbance trace of the size exclusion (right). On the traces, the green line in A. represents the % buffer B, the blue line shows the UV absorbance at 280 nm in arbitrary units; M: molecular marker, W: whole cell, S: soluble. (DOCX) [file pone.0206949.s002.docx]

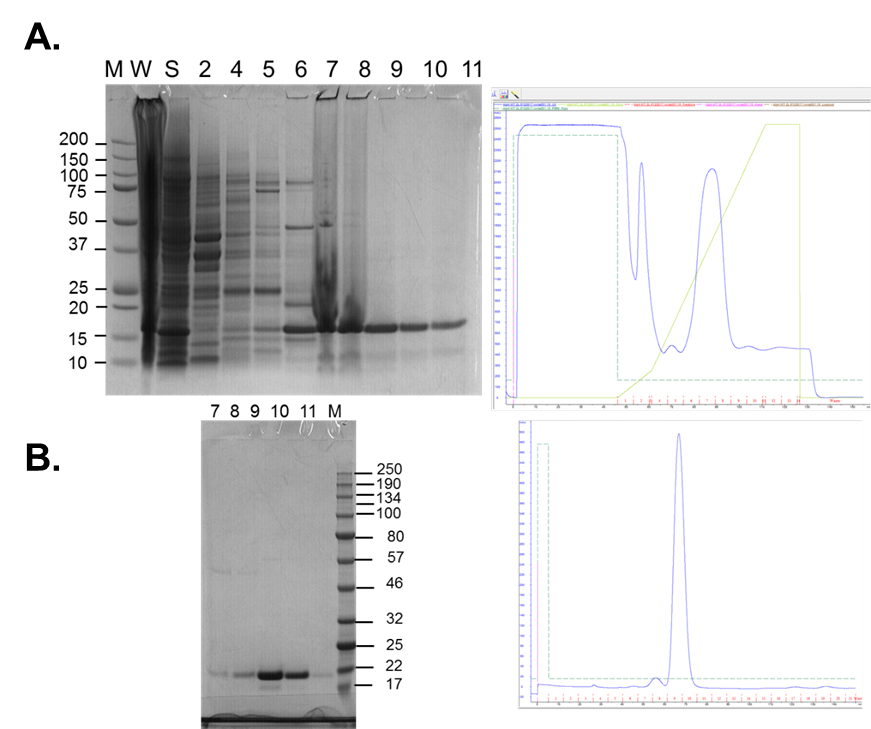


**S2 Fig: Purification of heterologous AtzH from *E. coli*.** A. SDS-PAGE analysis of the fractions 2-11 of the Ni-NTA purification of His_6_-AtzH (left), UV absorbance trace of the Ni-NTA purification (right), fractions 7 to 11 were concentrated and further purified; B. SDS-PAGE analysis of the size exclusion, showing the content of fractions 7-11 (left); UV absorbance trace of the size exclusion (right). On the traces, the green line in A. represents the % buffer B, the blue line shows the UV absorbance at 280 nm in arbitrary units; M: molecular marker, W: whole cell, S: soluble.
