# Supplementary material for: A novel decarboxylating amidohydrolase involved in avoiding metabolic dead ends during cyanuric acid catabolism in Pseudomonas sp. strain ADP
Source: PLoS One. 2018 Nov 6;13(11):e0206949. doi: 10.1371/journal.pone.0206949 (PMC6219798; doi:10.1371/journal.pone.0206949)
Supplement: S3 Fig — A. Bacteria containing an allantoate hydrolase and glutamyl-transferase enzymes upstream of the atzG-atzE-atzH segment; B. Bacteria found to be containing an allophanate hydrolase homologue of AtzF found upstream of the atzG-atzE-atzH segment; C. Bacteria containing a non-adjacent AtzH homologue, located between a monooxygenase and reductase enzymes, downstream of the atzG-atzE segment; D. Bacteria presenting the atzG-atzE-atzH cluster within unique gene organizations. A legend is shown at the top of the figure. Green arrows represents amidase type enzymes, black and grey tones represent ABC transporters, discontinued dark blue arrows represent the presence of a LysR type regulator, genes encoding for AtzG, AtzE and AtzH homologues are shown by orange, yellow and pink arrows and light blue arrows represent other types of enzymes, respectively. This figure is not to scale. (DOCX) [file pone.0206949.s003.docx]

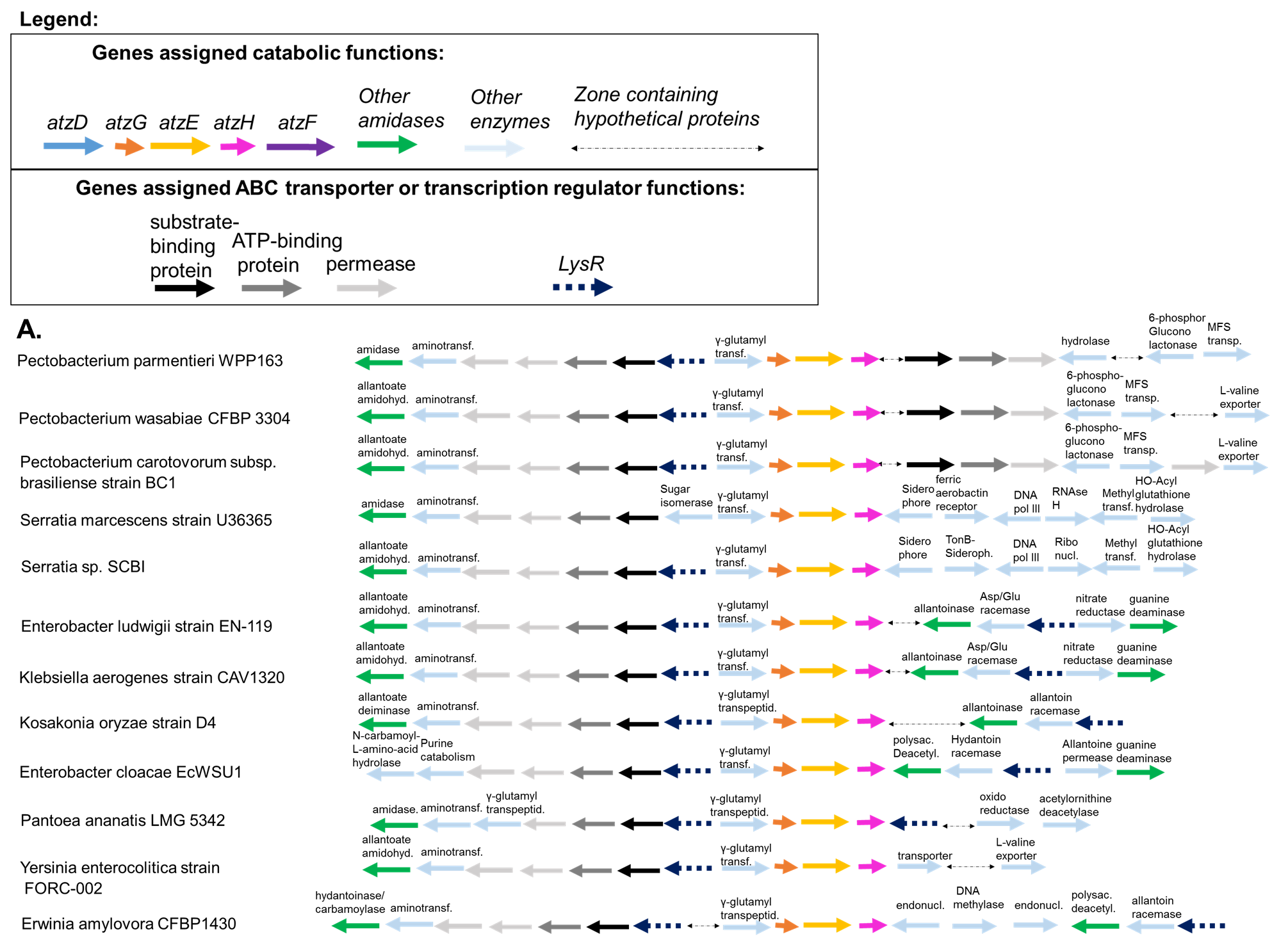

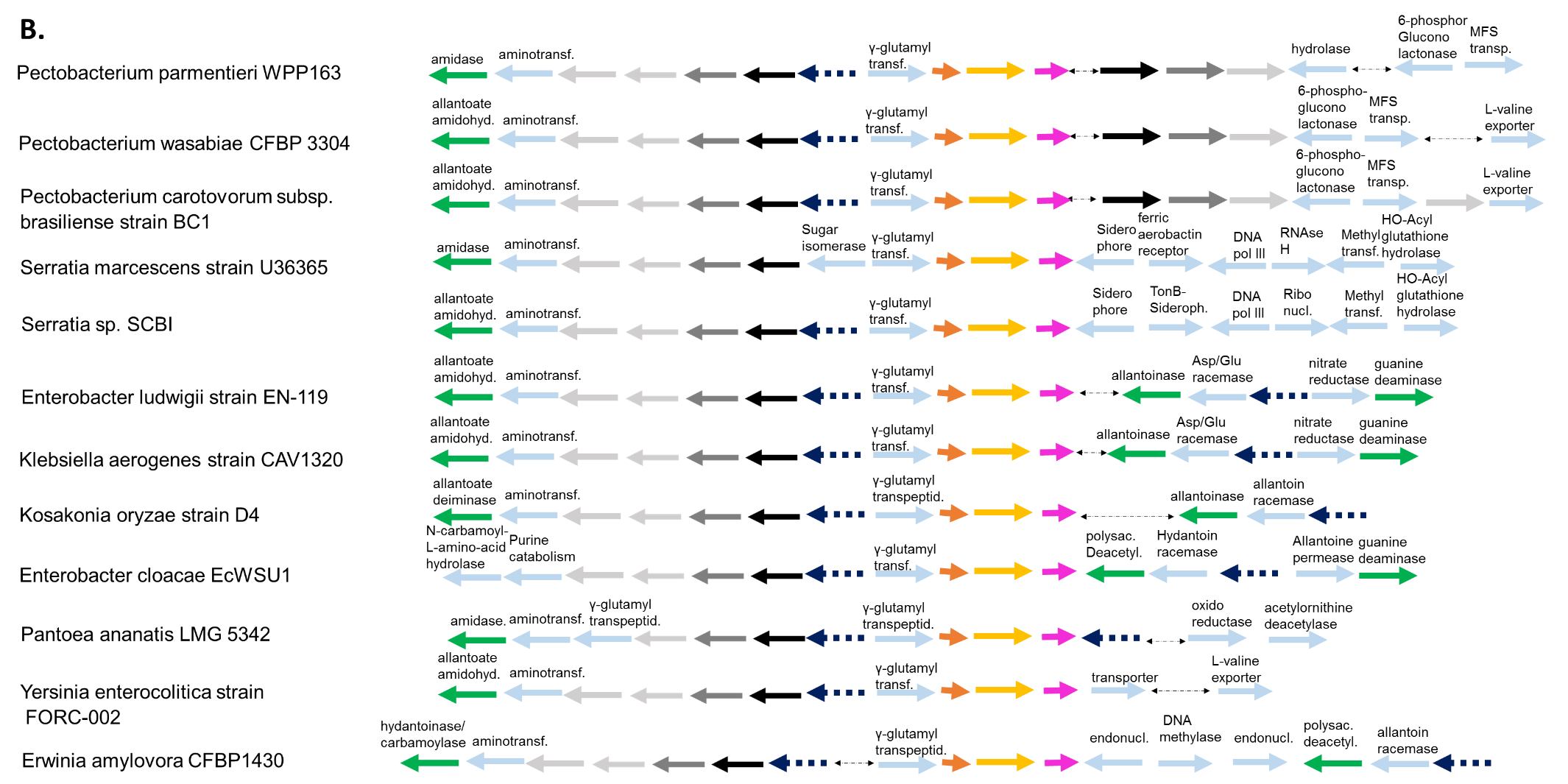

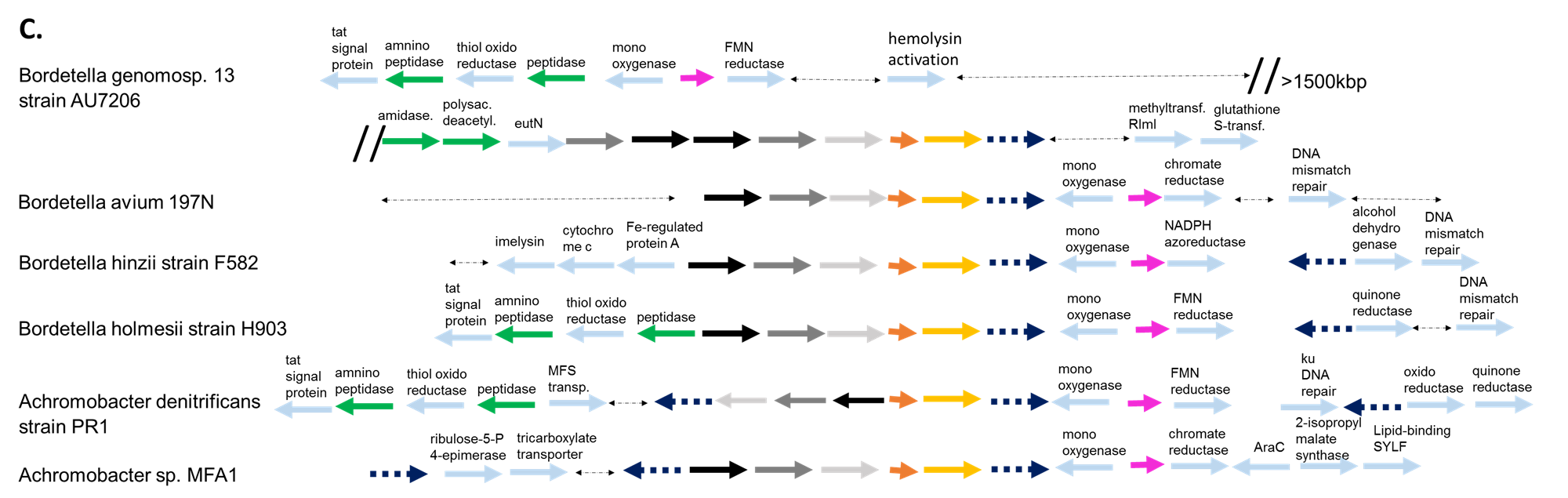

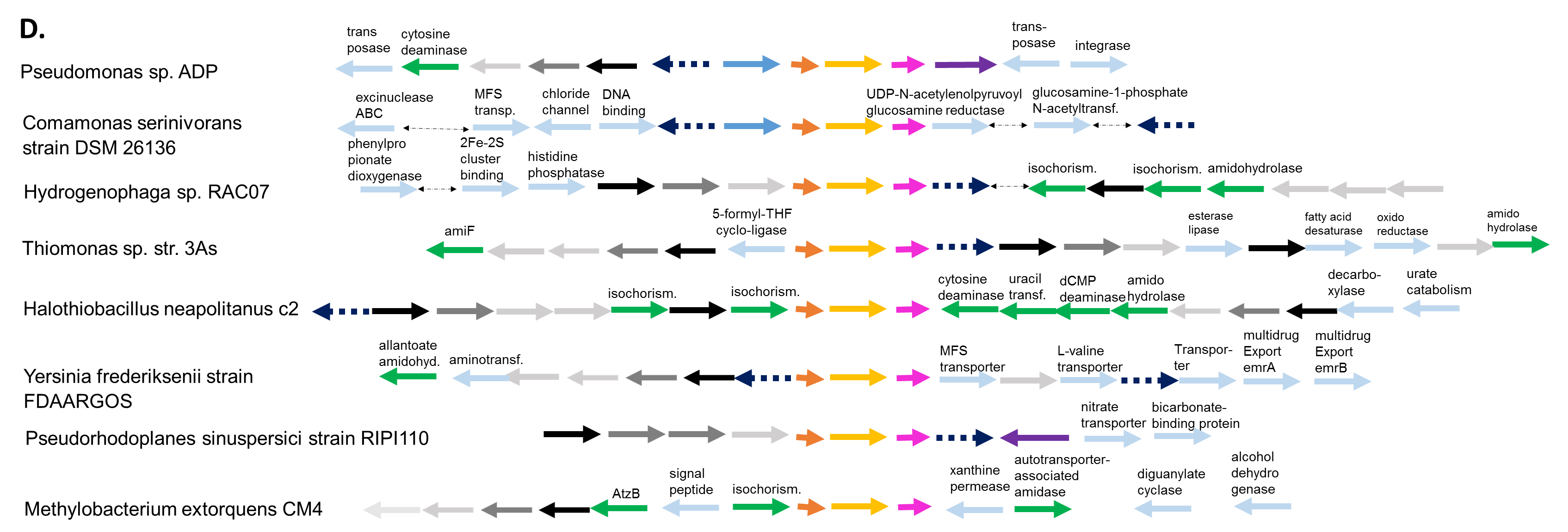


**S3 Fig: Genetic organization found flanking *atzE* homologues in other types of bacteria.** A. Bacteria containing an allantoate hydrolase and glutamyl-transferase enzymes upstream of the *atzG*-*atzE*-*atzH* segment; B. Bacteria found to be containing an allophanate hydrolase homologue of AtzF found upstream of the *atzG*-*atzE*-*atzH* segment; C. Bacteria containing a non-adjacent AtzH homologue, located between a monooxygenase and reductase enzymes, downstream of the *atzG*-*atzE* segment; D. Bacteria presenting the *atzG*-*atzE*-*atzH* cluster within unique gene organizations. A legend is shown at the top of the figure. Green arrows represents amidase type enzymes, black and grey tones represent ABC transporters, discontinued dark blue arrows represent the presence of a *LysR* type regulator, genes encoding for AtzG, AtzE and AtzH homologues are shown by orange, yellow and pink arrows and light blue arrows represent other types of enzymes, respectively. This figure is not to scale.
