# Supplementary material for: A novel decarboxylating amidohydrolase involved in avoiding metabolic dead ends during cyanuric acid catabolism in Pseudomonas sp. strain ADP
Source: PLoS One. 2018 Nov 6;13(11):e0206949. doi: 10.1371/journal.pone.0206949 (PMC6219798; doi:10.1371/journal.pone.0206949)
Supplement: S1 Table — (DOCX) [file pone.0206949.s004.docx]

**S1 Table:** **Mutagenic primers used for introducing point mutation in AtzH’s sequence.**

| **Amino acid mutated** | | **Mutagenic primer 5’ 🡪 3’** |
| --- | --- | --- |
| Tyr22Ala Fwd | cgcccaattcgttcgcgccgagaaagcgcttacc | |
| Tyr22Phe Rev | gcgctttctcgaagcgaacgaattgggcg | |
| Arg46Ala Fwd | cccccaaaccctggcctacggtgcgacc | |
| Arg46Lys Fwd | agcccccaaaccctgaagtacggtgcgaccgag | |
| Arg63Ala Fwd | gcgtgtggcggcaaagcctgcaatggcctca | |
| Arg60Lys Rev | agagcgtgtggccttaaagcctgcaatggcctcatagc | |
| Arg66Ala Fwd | aggctttcgcgccacagcctctccaaacaacttg | |
| Arg63Lys Rev | ccaagttgtttggagactttgtggcgcgaaagcctgcaatgg | |
| Arg73Ala Fwd | gctctccaaacaacttggaagccgagatcgtccgga | |
| Arg73Lys Rev | ccgtccggacgatctccttttccaagttgtttggagagcgt | |
| Arg96Ala Fwd | ccgccaacatcgaattccgtgcgctcagtcatagtc | |
| Arg96Lys Fwd | taccgccaacatcgaattccgtaagctcagtcatagtcag | |
| Glu106Ala Fwd | gcttacgggccgggcgagccaaacctgg | |
| Glu106Ala Rev | gggccggcagagcgcaacctggatgcgc | |
