# Supplementary material for: A novel decarboxylating amidohydrolase involved in avoiding metabolic dead ends during cyanuric acid catabolism in Pseudomonas sp. strain ADP
Source: PLoS One. 2018 Nov 6;13(11):e0206949. doi: 10.1371/journal.pone.0206949 (PMC6219798; doi:10.1371/journal.pone.0206949)
Supplement: S2 Table — (DOCX) [file pone.0206949.s005.docx]

**S2 Table: DSF of the AtzH variants.**

| **AtzH Variant** | **T_m_ (°C)** |
| --- | --- |
| Y22A | 48.8 +/- 0.04 |
| Y22F | 57.0 +/- 0.09 |
| R46A | 55.3 +/- 0.02 |
| R46K | 58.3 +/- 0.05 |
| R63A | 55.3 +/- 0.04 |
| R63K | 54.2 +/- 0.13 |
| R66A | 55.3 +/- 0.04 |
| R66K | 55.0 +/- 0.05 |
| R73A | 55.5 +/- 0.06 |
| R73K | 50.6 +/- 0.09 |
| R96A | 57.0 +/- 0.03 |
| R96K | 55.6 +/- 0.05 |
| Q106A | 50.2 +/- 0.04 |
| Q108A | 52.1 +/- 0.06 |
